# The Efficacy of On-Site Integration Screening and Microelimination Programs for Chronic Hepatitis C in a Detection Center: A Comparison of the Treatment Outcomes and Characteristics of Incarcerated Patients and Outpatients

**DOI:** 10.1155/2024/3184892

**Published:** 2024-03-13

**Authors:** Hsuan-Yuan Chang, Su-Hung Wang, Hsing-Tao Kuo, Ming-Jen Sheu, I-Che Feng, Chung-Han Ho, Jui-Yi Chen, Chi-Shu Sun, Chi-Hsing Chen, Cheng-Yi Lin, Chun-Chi Yang

**Affiliations:** ^1^Division of Hepatogastroenterology, Department of Internal Medicine, Chi Mei Medical Center, Tainan, Taiwan; ^2^School of Medicine, College of Medicine, National Sun Yat-sen University, Kaohsiung, Taiwan; ^3^Department of Medical Research, Chi Mei Medical Center, Tainan, Taiwan; ^4^Department of Information Management, Southern Taiwan University of Science and Technology, Tainan, Taiwan; ^5^Division of Nephrology, Department of Internal Medicine, Chi Mei Medical Center, Tainan, Taiwan; ^6^Department of Health and Nutrition, Chia Nan University of Pharmacy and Science, Tainan, Taiwan

## Abstract

We aimed to analyze the different patient characteristics and treatment outcomes (such as sustained viral response, SVR) between incarcerated patients with chronic hepatitis C (CHC) and those with CHC from the outpatient department through an on-site integrated screening and microelimination program in a detection center. In this retrospective study, which ran from May 2021 to April 2022, we included 32 consenting male prisoners aged at least 20 years who were willing to participate in the study. Members of the control group (who received DAAs in an outpatient setting) were selected from the treated CHC patient databank of individuals who received DAA regimens at Chi Mei Hospital between January 2021 and December 2022. The patients in the two groups did not differ significantly in terms of age, FIB-4 score, HCV RNA, HBV coinfection, hemogram findings, coagulation profiles, and renal function tests. However, the patients in the incarcerated group had a significantly different genotype distribution compared to the control group, significantly lower liver enzyme levels, and higher albumin and bilirubin levels compared to those in the control group. The rate of SVR to DAA treatment obtained among incarcerated patients did not differ significantly from that obtained among patients in the control group. Loss to follow-up (for several reasons) is a major reason for treatment discontinuation among these patients.

## 1. Introduction

Chronic hepatitis C virus (HCV) infection is a known global health problem, prompting the World Health Organization (WHO) to define elimination goals by 2030 by reducing new infections by 90% and mortality by 65% [1]. People incarcerated in prisons or jails are a key risk group for HCV infection. The prevalence of HCV antibodies (Ab) in closed settings was 30% in Western Europe, as estimated in 2013 by Larney et al. This prevalence varies worldwide as follows: approximately 35% in Australia, 29% in North America, and 25% in East Asia [2]. The HCV prevalence among people in correctional facilities is not geographically uniform and can vary by state and region [3]. At any given moment, an estimated 10.7 million incarcerated people worldwide constitute a key group to target for HCV elimination [4]. In Taiwan, the prevalence of anti-HCV Ab seropositivity was 33.5% and 34.8% in two trials conducted in recent years [5, 6]. Injection drug use is the most common risk factor for HCV transmission in correctional settings [7, 8]. Tattooing, sharing of the injecting apparatus, risky sexual behavior, barbering, and even fighting were all transmissive methods identified among incarcerated individuals with HCV infection [9, 10]. These were the main reasons why incarcerated individuals had a high prevalence of chronic hepatitis C (CHC). HCV-associated liver disease, a frequent cause of death among inmates, has recently surpassed HIV in this respect [11]. Several preventive strategies and measures to block possible transmission routes of HCV, such as increasing knowledge and awareness of the prevention and auditing the competence of healthcare facilities, folk therapists, and tattoo artists on infection control procedures, were proposed [12]. However, due to the high efficacy of novel direct-acting antiviral agent (DAA) development, one of the most efficient methods of preventing transmission may be CHC eradication via the use of DAAs [13].

Pangenotype DAAs, which have been reimbursed by Taiwan National Health Insurance (NHI) since 2017, have become the treatment modality for CHC because of their high sustained viral response (SVR) rates in the general population. In the 2020 Taiwan consensus for special populations, many clinical trials and accumulating real-world data suggest that DAA regimens are effective and safe in patients who inject drugs (PWID) [14]. Moreover, DAAs are also preferred in custodial settings because of their shorter treatment course, high effectiveness, and fewer adverse effects than pegylated interferon plus ribavirin. DAA therapy for HCV is now logistically feasible within the prison setting and actually aids HCV elimination [15].

Places with a high prevalence of HCV, such as prisons, have been recognized as potential settings to implement interventions aimed at achieving HCV microelimination [16]. As the TraP HepC study conducted in Iceland demonstrates, screening and simultaneous treatment for CHC were offered to all incarcerated individuals. Such microelimination programs in high-prevalence populations can help nations achieve the goal of HCV elimination [17]. Thus, this case-control study is aimed at analyzing the different patient characteristics and treatment outcomes (SVR) between incarcerated CHC patients (incarcerated group) and CHC patients from the outpatient department (outpatient group).

## 2. Materials and Methods

### 2.1. Methods

Through the on-site integrated screening and microelimination program in a detection center in collaboration with the correctional facility HCV screening and treatment program elaborated by the National Health Insurance Administration and Public Health Bureau, our hospital began implementing an on-site integrated screening and elimination program in the Tainan detection center in April 2021. In the initial phase of the program, we provided group health education lectures to incarcerated persons and obtained written informed consent prior to screening. Mass screening was conducted by an outreach blood test service during a two-week period for all incarcerated persons to check for anti-HCV seropositivity. Reflex HCV ribonucleic acid (RNA) examination was performed spontaneously. All incarcerated persons in whom HCV RNA was detected were offered the opportunity to undergo further evaluation and receive treatment at a special on-site hepatitis C clinic. To address this program, we established and operated a special on-site hepatitis C clinic in the Tainan detection center on a weekly basis after mass screening for HCV infection. The clinic has two hepatologists, one registered nurse, and one case manager in its staff.

**Figure 1:**
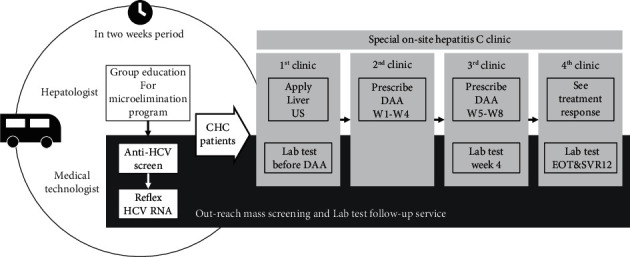
The on-site integrated screening and microelimination program in a detection center. Abbreviations: HCV: hepatitis C virus; CHC: chronic hepatitis C; US: ultrasound; Lab: laboratory; DAA: direct antiviral agent; EOT: end of treatment; SVR: sustained viral response.

To facilitate complete pre-DAA treatment assessment during the first visit to the clinic, a portable ultrasound machine for abdominal ultrasonography was equipped in the prison clinic and all necessary samples for laboratory tests before DAA treatment were collected at the same time. Patients who tested positive for HCV RNA were treated with glecaprevir/pibrentasvir (GLE/PIB) or sofosbuvir/velpatasvir (SOF/VEL) if liver decompensation was detected upon presentation at the clinic or anamnesis. All treatment and follow-up strategies were performed according to the Taiwan NHI clinical practice guidelines. We implemented a facilitated and streamlined process to reduce the treatment duration and minimize the number of visits to the clinic. Patients treated with GLE/PIB required only four clinic visits to complete treatment and all necessary follow-up (Figure 1).

### 2.2. The Study Design

The on-site integrated screening and elimination program was scheduled and initiated since May 1, 2021. However, this program was disrupted by the COVID-19 pandemic just after the mass screening was completed. Therefore, the first clinic for treatment was established in August 2021.

This retrospective study was conducted between May 1, 2021, and April 30, 2022, to evaluate the efficacy of DAA therapy and the epidemiology of HCV infection in incarcerated individuals at a detection center. Only patients with chronic HCV infection who completed a full course of DAA treatment were included in the analysis to ensure data integrity. To compare characteristics between incarcerated individuals with CHC and community-dwelling individuals with CHC, a control group was included in this study. The control group consisted of CHC patients who received DAA therapy at Chi Mei Hospital, a tertiary medical center in Tainan, between January 1, 2021, and December 31, 2022.

The Institutional Review Board (IRB) of Chi Mei Hospital approved this study under the approval number 11208-001, and the confidentiality of enrolled patients was protected according to the principles of the Declaration of Helsinki and the International Conference on Harmonization for Good Clinical Practice.

### 2.3. HCV-Viremic Patients Identified by Mass On-Site Integrated Screening (Incarcerated Group)

All incarcerated individuals at the Tainan detection center (Agency of Corrections, Ministry of Justice, Taiwan) were invited to participate in the elimination program. We included all consenting male prisoners aged at least 20 years who were willing to participate in the study. Patients with less than six months remaining on their sentences were excluded from this study as they would have been released from jail before the end of the study period. Patients who had severe illness or hepatic decompensation were also excluded from this study. Additionally, patients who had previously received DAA treatment were excluded based on the treatment criteria of the NHI. Incarcerated individuals who tested positive for HCV RNA were defined as CHC-viremic patients, and they were referred to the special on-site hepatitis C clinic for treatment. Only patients who completed the treatment and reached the end-of-treatment (EOT) point were included in the incarcerated group.

### 2.4. The Control Group and Propensity Score Matching

Members of the control group were selected from the treated CHC patient databank of individuals who received DAA regimens at Chi Mei Hospital between January 2021 and December 2022. To minimize the impact of selection bias, each incarcerated patient was matched with two controls using the propensity score matching approach on age and creatinine levels. Per the matching criteria, we identified 74 patients who met the matching criteria and were included as control participants for our analysis.

### 2.5. Laboratory Investigations

Demographic and baseline characteristics including sex, age, HCV genotype, HCV RNA viral load, coinfection with hepatitis B, hemogram (complete blood cell count), coagulation profile, hepatic and renal functions, and alpha-fetoprotein (AFP) levels were collected. Fibrosis-4 (FIB-4) scores were also calculated. Advanced hepatic fibrosis (fibrosis stage F3) was assessed using the fibrosis index based on the FIB-4 test, with a cutoff value of ≥3.25. Liver cirrhosis was diagnosed based on the presence of clinical, radiological, and endoscopic findings that met the criteria for cirrhosis. Patient treatment options were also recorded.

HCV genotypes (GTs) were analyzed via primer-specific polymerase chain reaction, and GTs were determined using the Abbott RealTime HCV Genotype II (Roche Molecular Diagnostics) with the Abbott m2000 system. To assess the virologic response after DAA therapy, serum HCV RNA levels were quantified using the Abbott RealTime HCV viral load assay (Abbott Molecular, USA) at two time points, EOT, and 12 weeks posttreatment (SVR12). The lower limit of detection was 15 IU/mL. Abdominal ultrasonography was performed prior to treatment initiation to detect the presence of liver cirrhosis and for hepatocellular carcinoma surveillance. Baseline laboratory tests were conducted within three months before treatment initiation.

### 2.6. Statistical Analysis

Statistical Analysis System (SAS) statistical software (version 9.4; SAS Institute, Inc., Cary, NC, USA) was used for all analyses. Continuous variables are presented as mean values (standard deviations) or median values (IQ1, IQ3), while categorical variables were presented as counts and percentages. Pearson's chi-square test or Fisher's exact test (categorical variables) and Student's *t* test or the Wilcoxon rank-sum test (continuous variables) were used to compare baseline features between incarcerated patients and control participants. A *p* value of < 0.05 was considered statistically significant.

## 3. Results

### 3.1. Patient Flowchart of the HCV Microelimination Program

**Figure 2:**
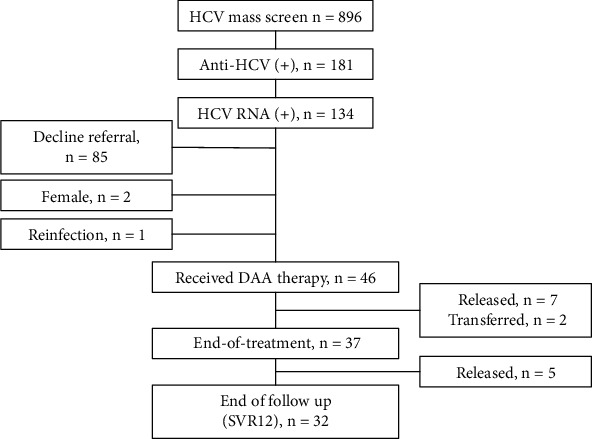
The patient flowchart of HCV mass screening. Abbreviations: HCV: hepatitis C virus; CHC: chronic hepatitis C; DAA: direct antiviral agent; SVR: sustained viral response.

Out of 1080 inmates, 896 (83.0%) participated in the mass screening, among whom 181 (20.2%) were anti-HCV seropositive. Of them, 134 (74.0%) tested positive for HCV RNA. However, 85 subjects declined referral to the special on-site hepatitis C clinic for reasons such as their unwillingness to undergo treatment, transfer to other jails, or imminent release. One patient was excluded due to retreatment for reinfection, and two female patients were also excluded. Ultimately, 46 (34.4%) patients received DAA treatment. However, seven patients were released from jail, and two patients were transferred to other correctional facilities during the treatment period. Ultimately, only 37 (80.4%) patients completed the treatment and reached the EOT point, and they were enrolled as the incarcerated group. In this group, five patients were released during the posttreatment follow-up period and 32(86.5%) patients reached the end of follow-up point (SVR12). The patient flowchart of HCV mass screening, assessment, and treatment is presented in Figure 2.

### 3.2. Patient Characteristics

The baseline characteristics of patients in the incarcerated and control groups are shown in Table 1. Those in the incarcerated group had a mean age of 47 years, with only 2 (5.4%) patients having HBV coinfection. Only 2 (5.4%) of the patients in the incarcerated group had advanced fibrosis (with FIB-4 score of >3.25) and liver cirrhosis confirmed via ultrasonography. The mean HCV RNA level was 6.0 (log10 IU/mL), and the dominant HCV genotype was genotype 1 (GT1a+1b, 37.8%), followed by HCV-GT6 (18.9%), HCV-GT3 (16.2%), and mixed or indeterminate genotypes (16.2%).

The two groups did not differ significantly in terms of age, FIB-4 score, HCV RNA, HBV coinfection, hemogram findings, coagulation profiles, or renal function tests. However, the patients in the incarcerated group had a significantly different genotype distribution compared to the control group, significantly lower liver enzyme levels, and higher albumin and bilirubin levels compared to those in the control group. None of the patients had decompensated cirrhosis or liver cancer.

### 3.3. Treatment Efficacy

**Figure 3:**
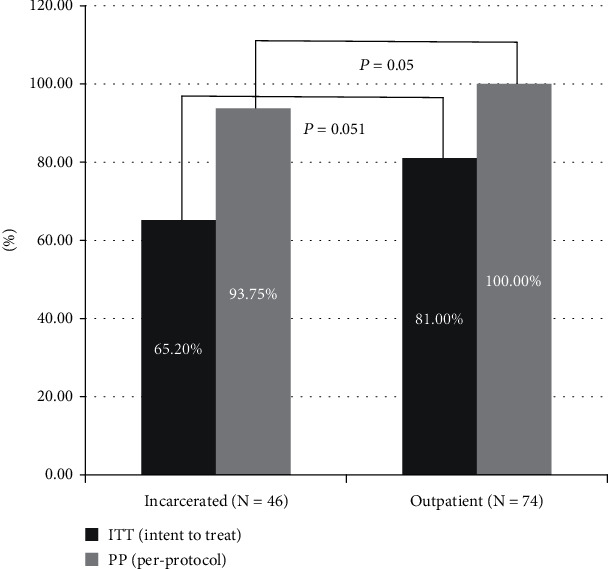
The SVR of DAA therapy between the incarcerated and outpatient HCV patients. Abbreviations: ITT: intention-to-treat; PP: per-protocol.

In the incarcerated group, 46 patients received DAA treatment and only 37 of them completed the treatment course, with SVR12 being achieved in 32 of them. Nine patients (seven who were released early and two who were transferred to other prisons) during the period of treatment and five during the follow-up period were lost to follow-up in the incarcerated group. Fourteen patients were also lost to follow-up in the control group. The rate of intention-to-treat (ITT) SVR was 65.2% in the incarcerated group and 81.0% in the control group. Lower ITT SVR was achieved among incarcerated patients; however, the difference was not statistically significant (65.2% vs. 81.0%, *p* = 0.051). The per-protocol (PP) SVRs were 93.8% and 100% in the incarcerated and outpatient groups, respectively; however, the difference between them was not statistically significant (*p* = 0.05; Figure 3).

## 4. Discussion

Every year, over 10 million individuals worldwide, both men and women, are confined to prisons and other closed facilities. Approximately 30% of all persons with HCV infection in the US have spent at least a fraction of a year in a correctional institution [3]. However, only a few HCV-infected individuals in correctional facilities were aware of their infection [18]. The majority of incarcerated individuals will eventually be released and reintegrate into the general population, where they can contribute to the spread of HCV in the community [19]. HCV elimination strategies can help mitigate the financial and medical burdens caused by HCV. The success of these strategies relies not only on the efforts made within correctional settings but also on the collaboration of neighboring communities [20]. The US Preventive Services Task Force [21] and the WHO [22] recommend that all incarcerated persons undergo HCV testing. Despite these recommendations, HCV testing is still not universally performed at correctional institutions as a routine entrance examination in Taiwan. The primary reason for this observation is the payment issue within Taiwan's health insurance system. Reimbursement is not provided for routine examination for anti-HCV unless the individuals are identified as having chronic hepatitis, defined as abnormal liver function persisting for over 3 months. Therefore, even though DAA treatment can be reimbursed for all individuals with detected HCV-RNA under Taiwan's NHI, identifying and treating these potential HCV-infected patients in this high-risk population would not be achievable without the assistance of a special microelimination program funded by nongovernmental organizations (NGOs) to provide universal anti-HCV screening.

The microelimination approach, which focuses on targeting smaller, high-risk subpopulations for treatment, has been proposed as an effective way of addressing HCV infections [23]. The Australian experience from the Surveillance and Treatment of Prisoners with hepatitis C (SToP-C) study highlights the efficacy of access to CHC patients and engagement with CHC treatment for microelimination in incarcerated individuals [24]. Only a few single site studies with smaller samples have been conducted in Taiwan. Yang et al. arranged a microelimination program for individuals in prison in Taiwan. A total of 1,402 individuals were invited to participate in the screening and 59% of them accepted; 165 patients received DAAs, and the overall SVR12 rate was 100% [5]. Chen et al. arranged a HCV microelimination program in a prison in Taiwan populated mainly by PWID. Out of the 1,697 individuals invited to participate in anti-HCV mass screening, 1,137 (67%) accepted [6]. In our study, we observed a higher participation rate compared with the previous studies, with 83.0% of individuals willingly accepting screening. This was attributed to the on-site integrated screening program conducted by group education about this disease and the benefit of treatment, combined with an outreach blood test service over a two-week period for all incarcerated persons.

Data from prison-based treatment programs indicate that providing treatment to incarcerated individuals leads to positive clinical outcomes and is cost-effective [25–27]. These successful programs can serve as a guide for stakeholders, including the government, in implementing HCV elimination programs both in correctional facilities and in the general population.

The incarcerated population was identified as a priority for microelimination efforts due to their high prevalence of CHC. Additionally, this group of incarcerated patients with HCV has certain characteristics that differentiate them from the general population. In 2018, Crowley et al. conducted a qualitative study that revealed that the barriers to HCV screening and treatment included lack of knowledge, concerns regarding confidentiality, fear of stigmatization, liver biopsy phobia, inconsistent access to prison health services, and delays in screening and treatment reception [28]. However, the treatment cascade existed in care and treatment among patients in the incarcerated population. The SVR12 achievement rate was excellent under the strategies of ambulatory clinic or on-site screening and treatment program. In our study, the PP SVR in both groups exceeded 90%. The main reason why all patients did not achieve SVR was the loss to follow-up due to early release or transfer. The virus was not detected in the serum of any patient at the EOT.

The models of HCV care within correctional settings exhibit significant variation both within and between countries. In addition to the unique characteristics of incarcerated individuals with HCV, another issue may result in treatment interruption or discontinuation of follow-up. This issue pertains to early release or prison transfer, which is critical when implementing HCV treatment within correctional facilities. According to the results of our study, nine inmates failed to reach the EOT due to early release or prison transfer. This phenomenon was indirectly responsible for our finding of no significant difference in the ITT SVR between the incarcerated group and the control group. Upon release from prison and the subsequent loss of access to ongoing treatment and follow-up, incarcerated individuals enter a period of heightened vulnerability. During this time, they may encounter negative health effects stemming from the absence of long-term physical and mental support, including the potential for treatment failure and the risk of reinfection [29].

Previous studies have revealed that loss to follow-up upon early release from prison was a major obstacle to HCV treatment for incarcerated patients due to long waiting times when undergoing screening for indigent care services, difficulty obtaining medications after being released, and lack of awareness as to where and how to access further treatment [15, 30]. Retrospective studies conducted in the USA have indicated that only 10% of individuals who were previously incarcerated manage to establish a connection with HCV care after their release [31, 32]. Brief periods of incarceration result in significant rates of treatment discontinuation, emphasizing the critical need to ensure continuity of care following release [33]. To address the critical challenges facing the linkage of individuals to care, particularly during transfers between correctional settings and short stays, it may be worthwhile to explore more streamlined and tailored models of HCV care for implementation within correctional settings. There are a variety of interventions that can be implemented to support released prisoners with linkages to care. Research on HIV has found that arranging an appointment with an HIV primary care provider for released patients before their release increases the likelihood that they will complete their postrelease follow-up [34]. Similar interventions for supporting linkage to care should also be applied to HCV care. Improved models of HCV care can be sustained through the incorporation of in-reach services, where clinicians visit correctional centers to provide on-site clinic sessions. Hariri et al. conducted a comprehensive HCV care model in an Iranian provincial prison and found that over two-thirds of patients have demonstrated the ability to be connected to care after their release, underscoring the crucial role of active patient navigation in engaging individuals with postrelease care [35]. Additionally, it may be worthwhile to consider the integration of telemedicine consultations, which have been demonstrated to be both cost-effective and well-received [20]. The AASLD guidelines also recommend providing linkage to community healthcare upon discharge from correctional settings [36]. Community-based organizations and nongovernmental organizations can play a vital role in facilitating the successful community reintegration of offenders [37]. The New York State Hepatitis C Continuity Program provided evidence that establishing a network of community-based providers is feasible and effective in ensuring the uninterrupted continuation of HCV treatment following release [38].

Based on the characteristics of the two groups of patients in our study, significant distribution differences were observed, including the GT of HCV. The control group had the highest prevalence of GT 2, while the incarcerated group had the highest prevalence of GT 1 (1a+1b). This finding is consistent with that of a meta-analysis conducted in 2022 [39]. Furthermore, as mentioned earlier, it was initially expected that the ITT rate in the incarcerated group would be significantly higher than that in the control group due to characteristics such as shorter sentences and treatment duration (with the incarcerated group having a significantly higher rate of GLE/PIB usage than the control group), higher medication adherence, and better monitoring. However, due to the limited sample size of the incarcerated group in our study and the issue of early release or prison transfer before the achievement of SVR12 during the follow-up period, the ITT analysis did not show significant differences despite certain findings coming close to being significantly lower in the incarcerated group than in the control group.

This study had several limitations. First, it was a retrospective study that may have included biases such as researcher bias or information bias. Second, the sample size of incarcerated patients in our study was relatively small, resulting in increased variability that could impact the precision and reliability of the study's findings. Third, we did not document the side effects of DAA therapy that may have contributed to the failure to achieve SVR. Furthermore, our study did not assess the cost of antiviral treatment, which precluded a cost-benefit analysis.

## 5. Conclusions

In conclusion, implementing a comprehensive on-site microelimination program in prison settings (including education programs, outreach screening, and treatment) appears highly viable. The emergence of DAA drugs undoubtedly brings great benefits to inmates affected by HCV. Except for uncontrollable factors such as early release and prison transfer, it is highly promising to eradicate HCV in the near future if the government, prisons, hospitals, and community organizations collaborate and strengthen postrelease follow-up and treatment continuity. This approach would effectively contribute to the achievement of WHO targets, the mitigation of the risk of advanced liver disease progression, and the extension of benefits to the entire community upon prisoners' release.

## Figures and Tables

**Table 1:** Baseline characteristics: sporadic outpatient HCV therapy (control group) vs. incarcerated patients (incarcerated group).

Characteristics	Control (*n* = 74)	Incarcerated (*n* = 37)	*p* value
Age (years)	49.0 (41.0, 58.0)	47.0 (42.0, 53.0)	0.4851
HCV genotype (%)			0.0008
1a	14 (18.92)	8 (21.62)	
1b	12 (16.22)	6 (16.22)	
2	30 (40.54)	4 (10.81)	
3	3 (4.05)	6 (16.22)	
6	14 (18.92)	7 (18.92)	
Mixed	0	4 (10.81)	
Indeterminate	1 (1.35)	2 (5.41)	
Fibrosis-4 score (%)			0.2234
F0	32 (43.24)	22 (59.46)	
F1	17 (22.97)	10 (27.03)	
F2	11 (14.86)	2 (5.41)	
F3	9 (12.16)	1 (2.70)	
F4	5 (6.76)	2 (5.41)	
Liver cirrhosis (%)	3 (4.02)	2 (5.41)	1
HCV RNA (log10 IU/mL)	6.2 (5.3, 6.6)	6.0 (5.2, 6.4)	0.0831
HBsAg+ (%)	6 (8.11)	2 (5.41)	0.7165
WBC (1000/u)	5.8 (4.9, 7.2)	6.1 (5.5, 7.8)	0.4546
Hemoglobin (g/dL)	14.7 (13.3, 15.8)	15.3 (14.4, 16.2)	0.0929
Platelet (1000/u)	194.5 (158.0, 235.0)	198.0 (172.0, 232.0)	0.8267
Creatinine (mg/dL)	0.9 (0.8, 1.0)	0.9 (0.8, 1.0)	0.9897
PT (seconds)	11.6 (11.0, 11.9)	11.3 (11.1, 11.8)	0.3687
INR	1.0 (1.0, 1.1)	1.0 (1.0, 1.1)	0.824
Albumin (g/dL)	4.3 (4.0, 4.5)	4.4 (4.2, 4.6)	0.0128
ALT (IU/L)	55.0 (40.0, 149.0)	38.0 (26.0, 64.0)	0.0028
AST (IU/L)	40.5 (29.0, 77.0)	30.0 (23.0, 44.0)	0.0026
Total bilirubin (mg/dL)	0.7 (0.5, 0.9)	1.0 (0.7, 1.3)	0.0004
Direct bilirubin (mg/dL)	0.3 (0.2, 0.4)	0.4 (0.3, 0.5)	0.0223
AFP (ng/mL)	5.9 (4.0, 8.9)	5.1 (4.3, 8.0)	0.5948
Treatment options (%)			<0.0001
Epclusa (sofosbuvir/velpatasvir)	47 (63.51)	8 (21.62)	
Maviret (glecaprevir/pibrentasvir)	27 (36.49)	29 (78.38)	

Data are presented as the median (IQ1, IQ3). Categorical variables were reported as counts and percentages. Abbreviations: HCV: hepatitis C virus; HBV: hepatitis B virus; RNA; ribonucleic acid; ALT: alanine aminotransferase; AST: aspartate aminotransferase; WBC: white blood cell; AFP: alpha-fetoprotein.

## Data Availability

The data that support the findings of this study are available upon reasonable request from the corresponding author.
